# Posterior Release Surgery Further Improves the Efficacy of Halo-Pelvic Traction in the Treatment of Patients With Severe Spinal Deformity

**DOI:** 10.7759/cureus.99529

**Published:** 2025-12-18

**Authors:** Pengfan Song, Chiying Du, Zhong Zhang, Yi-Jian Liang

**Affiliations:** 1 Urology, Chengdu Third People's Hospital, Chengdu, CHN; 2 Anesthesiology, Guangxi Medical University Affiliated Tumor Hospital, Nanning City, CHN; 3 Orthopedics, Second Affiliated Hospital of Chongqing Medical University, Chongqing, CHN; 4 Orthopedics, BOE Chengdu Hospital, Chengdu, CHN

**Keywords:** halo-pelvic traction (hpt), posterior release surgery, scoliosis correction rate, severe spinal deformity, spinal scoliosis correction

## Abstract

Objective: To explore the effect of posterior release surgery combined with halo-pelvic traction in improving spinal deformity and to evaluate the factors affecting the correction rate of scoliosis.

Background: Spinal surgery is the preferred treatment for severe scoliosis and kyphosis, but direct primary corrective surgery carries significant risks and demands. Traction surgery has been shown to improve safety and spinal correction rates. However, traction surgery has a ceiling effect. In recent years, posterior release surgery has emerged as an emerging technique for spinal correction, but further research is needed to improve correction rates for severe scoliosis.

Methods: Fifty-four patients with spinal deformity treated between April 2021 and December 2023 were divided into two treatment phases: a halo-pelvic traction phase (Phase I) and a posterior spinal release followed by a halo-pelvic traction phase (Phase II). The effects of Phase I and Phase II on scoliosis and kyphosis correction were retrospectively evaluated, and statistical analysis was performed to identify factors influencing scoliosis correction rates.

Results: The first-stage scoliosis correction rate was 31.9%, and the kyphosis improvement rate was 23.1%. The second-stage scoliosis correction rate was 25.0%, and the kyphosis improvement rate was 19.5%. The overall scoliosis correction rate was 48.9%, and the kyphosis improvement rate was 38.0%. Statistical analysis showed that age, gender, and comorbidities (rib fusion or spinal fusion) were significant risk factors for scoliosis correction.

Conclusion: Posterior release surgery combined with halo-pelvic traction can significantly improve the correction effect of patients with severe spinal deformity.

## Introduction

Spinal deformity is a complex three-dimensional deformity that not only affects the patient's appearance and mental health but may also cause serious physiological dysfunction [1]. Severe spinal deformity usually refers to a deformity with a Cobb angle greater than 100° in the coronal or sagittal plane [2]. Patients with severe spinal deformity are more likely to suffer neurological impairment during surgery than those with mild or moderate deformity [3]. In addition, many patients with severe scoliosis often have impaired lung function, which makes them more susceptible to complications directly related to surgery or anesthesia [4-6]. Although current treatment options include conservative treatments (such as bracing) and surgery, these methods have limited effectiveness, and for patients with severe scoliosis, drastic corrective surgery may carry the risk of nerve damage [7]. Preoperative traction can significantly reduce surgical risks [8,9]. Preoperative traction mainly includes the following methods: Halo-femoral traction (HFT), halo-gravity traction (HGT) and halo-pelvic traction (HPT) [10-12], each of which has its own unique advantages and disadvantages. First, HGT is a commonly used preoperative traction method. Studies have shown that although HGT can provide a certain correction effect, it has a ceiling effect, that is, most of the correction occurs within the first 1-3 weeks of treatment, and traction for more than 3 weeks cannot further improve the correction effect [13]. This limitation restricts its use in patients with severe, rigid scoliosis.

In contrast, HPT is often used to correct severe spinal deformities and minimize complications due to its ability to provide strong and continuous traction [12-14]. However, although HPT has shown significant effects in severe patients, it also faces the problem of the ceiling effect. Posterior release surgery (i.e., Ponte osteotomy), as an important means of correcting spinal deformity, has shown lasting value and potential in the treatment of scoliosis [15]. Posterior release is generally considered safer and associated with fewer complications than traditional anterior release methods [16]. By combining posterior release surgery with HPT, it is possible to more effectively release the stiff portion of the spine and achieve a greater correction during surgery. The core innovation of this study lies in the discovery that HPT has a ceiling effect, and that release surgery can overcome this limitation, allowing patients to further reduce their Cobb angle before undergoing scoliosis correction surgery. This approach not only overcomes the limitations of single therapy but also provides a more effective and safe treatment option for patients with severe scoliosis.

## Materials and methods

Study subjects

A total of 65 patients with severe scoliosis were included in this study. According to the screening criteria, 54 patients (83.1%) were included in the final analysis, while 11 patients (16.9%) were excluded. The screening criteria were as follows: Inclusion criteria: (1) Age range: 10 to 50 years old. (2) Diagnostic criteria: diagnosed with severe scoliosis (Cobb angle > 100°). (3) Surgical requirements: preoperative halo-pelvic traction and only a single posterior release surgery. Exclusion criteria: (1) Insufficient Cobb angle: preoperative scoliosis Cobb angle < 100°. (2) Number of surgeries: no posterior release surgery or two or more posterior release surgeries. All subjects were from Chengdu Third People's Hospital and signed informed consent before participating in the study. This study complies with the standards and requirements of the Ethics Committee of Chengdu Third People's Hospital.

Cohort flowchart

**Figure 1:**
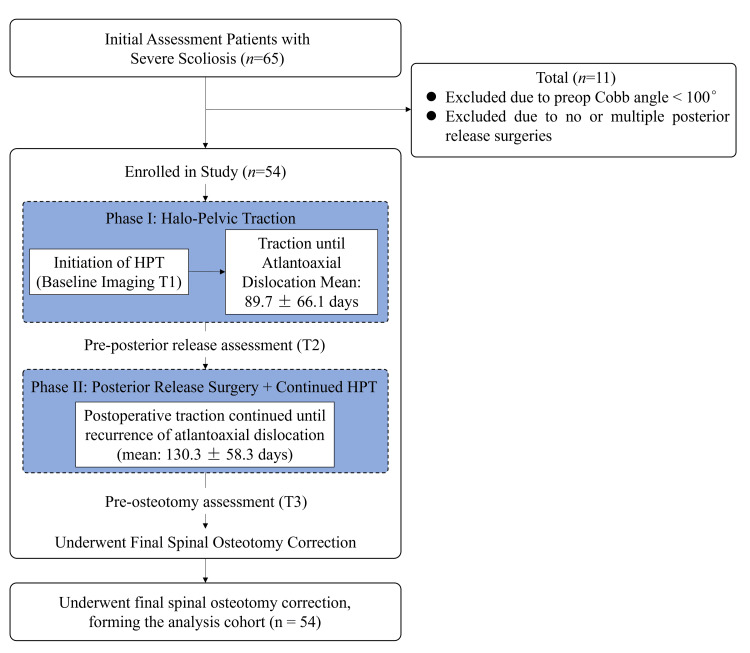
Study flowchart HPT: Halo-pelvic traction

The patient selection process is summarized in Figure 1. Initially, 65 consecutive patients with severe spinal deformity who were scheduled to undergo HPT between April 2021 and December 2023 were assessed for eligibility. Of these, 11 patients were excluded: 8 patients (72.7%) due to a preoperative Cobb angle of less than 100°, and 3 patients (27.3%) because they did not undergo the standardized single posterior release surgery as stipulated by the study protocol (two patients required an additional anterior release, and one patient proceeded directly to final correction without a posterior release). Consequently, a final cohort of 54 patients met all inclusion criteria and constituted the study population for retrospective analysis.

Study timeline

A visual representation of the study timeline and patient flow is provided in Figure 1. This retrospective study was conducted between April 2021 and December 2023. The patient recruitment and treatment period spanned this entire duration. Phase I (Halo-pelvic traction) was initiated upon enrollment and continued until the occurrence of atlantoaxial dislocation, with a mean duration of 89.7±66.1 days89.7±66.1days. Following Phase I, Phase II (Posterior release surgery followed by continued HPT)was performed, with traction maintained until atlantoaxial dislocation recurred, lasting a mean of 130.3±58.3 days. The final spinal osteotomy was then conducted upon completion of Phase II. Imaging assessments were performed at three critical timepoints: Pre-HPT (baseline), Pre-posterior release (post-Phase I), and Pre-final osteotomy (post-Phase II).

Interventions

Halo-pelvic Traction

Before HPT, patients undergo a comprehensive preoperative evaluation, including a physical examination and imaging studies (X-rays, CT scans, and MRIs) to determine the severity, type, and etiology of the scoliosis and to exclude contraindications. HPT is performed under general or local anesthesia, depending on the patient's condition and physical condition. The surgery utilizes a specific position: the patient is placed in the lateral position during pelvic pin insertion, with axillary pads placed to prevent shoulder compression and brachial plexus injury. The patient is placed in the supine position during head collar insertion. The pelvic pin insertion point is typically 2-3 cm above the anterior superior iliac spine, passing through four cortical layers before exiting at the posterior superior iliac spine. When the pelvic pin is placed on the other side, a foam pad is placed beneath the patient to ensure proper positioning. The head collar is secured to the ear margins and posterior brow arches: three screws are inserted 0.5 cm above the apex of each auricle, and two screws are inserted into each brow arch. To prevent postoperative eyelid closure problems, the patient's eyelids must be closed naturally during surgery. Postoperatively, halo-pelvic traction therapy is continued until the final osteotomy. During traction, traction parameters (such as traction force and direction) are adjusted according to the patient's weight and degree of scoliosis, and scoliosis improvement is regularly assessed.

Posterior Release Surgery

**Figure 2:**
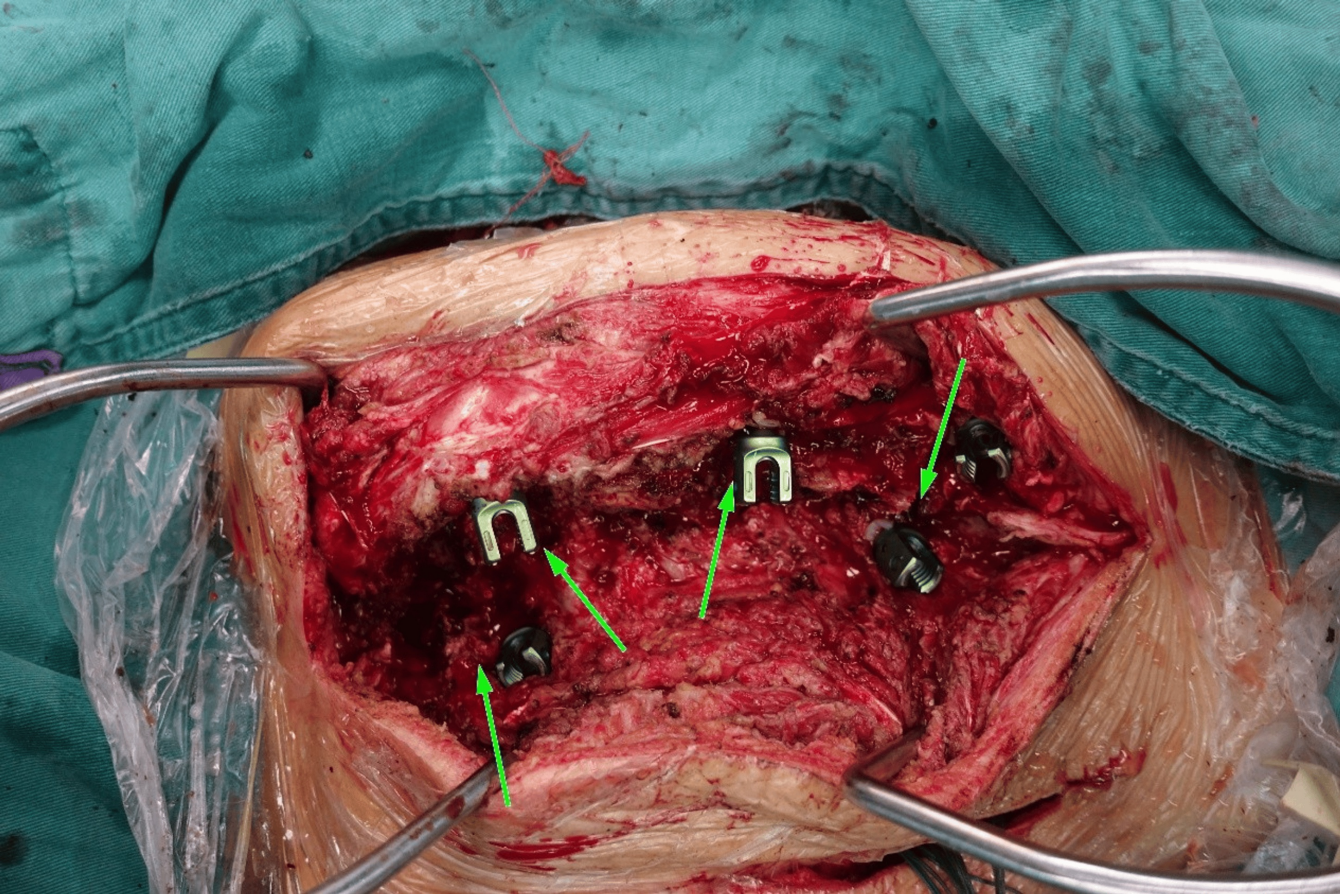
Posterior spinal release

After traction and fixation are complete (marked by the appearance of atlantoaxial dislocation), releases are performed at intervals of one vertebra, starting from the apex. This procedure involves partial resection of the supraspinal, interspinous, and flavum ligaments, as well as the superior and inferior articular processes. Screws are used to mark the specific release sites, reminding the patient to carefully approach the site during the next surgery to avoid spinal cord injury. After complete hemostasis, the incisions are sutured layer by layer and bandaged to prevent infection. Patients are observed for 6-7 days postoperatively. If there are no abnormalities such as intracranial, pelvic organ, or nerve damage, ambulation can be allowed. During the recovery period, HPT is continued until atlantoaxial dislocation recurs, indicating the end of the second traction session and preparation for osteotomy. Traction parameters (such as force and direction) are adjusted according to the patient's weight and degree of scoliosis, and scoliosis improvement is regularly assessed. Figure 2 shows the key steps of the intraoperative release procedure (arrows point to the four areas of intraoperative release).

Data collection

In order to evaluate the improvement of patients' deformity at different stages of treatment, this study set up three time points for imaging evaluation: (1) Before the HPT: full spine anteroposterior and lateral radiographs were taken to evaluate the initial spinal deformity. During the traction process, cervical spine anteroposterior and lateral radiographs were taken regularly, focusing on observing whether the atlantoaxial dislocation occurred. (2) Before the posterior release surgery: another imaging examination was performed to evaluate the initial effect of the HPT and observe whether the atlantoaxial dislocation occurred. (3) Before the spinal osteotomy correction surgery: the last imaging examination was performed to evaluate the overall effect of the combined treatment and observe whether the atlantoaxial dislocation occurred. All imaging data were measured using medical imaging software (INFINITT). The scoliosis Cobb angle and kyphosis Cobb angle were measured independently by two doctors, and the average value was taken as the final result.

Evaluation metrics

The evaluation indicators of this study include the patient's scoliosis Cobb angle and kyphosis Cobb angle. The specific measurement methods are as follows: Scoliosis Cobb angle: measured by measuring the inclination of the two vertebrae above and below the vertebrae at the apex of scoliosis. Kyphosis Cobb angle: measured by measuring the maximum curvature angle of the kyphotic part of the spine. In order to quantify the improvement effect, the "scoliosis correction rate" was introduced as an evaluation indicator, and its calculation formula is as follows:

$$\text{Scoliosis correction rate} = \frac{\text{preoperative Cobb angle} - \text{postoperative Cobb angle}}{\text{preoperative Cobb angle}} \times 100\%$$

This indicator reflects the degree of deformity improvement after the patient undergoes HPT and posterior release surgery [17].

Statistical analysis

This study used SPSS software for data analysis, with a significance level of p<0.05. Continuous variables were expressed as mean ± standard deviation, and categorical variables were expressed as counts and percentages. Analytical methods were as follows: Descriptive statistical analysis: Patient demographics, baseline characteristics, and clinical parameters were summarized. Inferential statistical analysis: Paired-sample t-tests were used to evaluate the therapeutic effects of cephalopelvic traction and posterior release. Patients were divided into a high-correction-rate group and a low-correction-rate group based on a mean curve correction rate of 48.9%. Further analyses: Chi-square tests were used to assess whether gender, curve type (congenital/idiopathic), comorbidities (rib fusion or spinal fusion), and spinal cord malformation influenced the correction outcome. Independent-sample t-tests were used to compare age, initial Cobb angle, and treatment duration between the two groups. Risk factor analysis: Binary logistic regression analysis was used to identify risk factors associated with the curve correction rate.

## Results

Patient clinical data and baseline characteristics

This study enrolled 54 patients with severe spinal deformity who underwent HPT and posterior release. The cohort consisted of 29 females (53.7%) and 25 males (46.3%); with a mean age of 26.1 ± 9.1 years and a mean BMI of 20.0 ± 3.8. Regarding scoliosis type, 39 patients (72.2%) had a congenital spinal deformity and 15 patients (27.8%) had an idiopathic spinal deformity. Concurrent rib or spinal fusion was present in 39 patients (72.2%) and absent in 15 patients (27.8%). A concurrent spinal cord deformity was present in 20 patients (37.0%) and absent in 34 patients (63.0%). The mean initial scoliosis Cobb angle was 140.3 ± 23.1 degrees, the mean initial kyphosis Cobb angle was 109.2 ± 34.8 degrees, and the mean treatment duration was 220.1 ± 94.4 days. Baseline clinical data are shown in Table 1.

**Table 1:** Baseline characteristics of the 54 patients Data are presented as mean ± standard deviation for continuous variables and as count (percentage) for categorical variables.

Characteristic	
Gender	
Male	25（46.3%）
Female	29（53.7%）
Age (years)	26.1±9.1
Body Mass Index (BMI)	20.0±3.8
Scoliosis Type	
Congenital	39（72.2%）
Idiopathic	15（27.8%）
Combined Rib Fusion or Spinal Fusion	
Yes	39（72.2%）
No	15（27.8%）
Combined Spinal Cord Malformation	
Yes	20（37.0%）
No	34（63.0%）
Initial Scoliosis Cobb Angle (degrees)	140.3±23.1
Initial Kyphosis Cobb Angle (degrees)	109.2±34.8
Treatment time (days)	220.1±94.4

Correction rate analysis in two phases

Table 2 shows the scoliosis and kyphosis correction rates for the 54 patients during the two treatment phases. The treatment duration for the first phase (from HPT to posterior release surgery) was 89.7 ± 66.1 days. The initial scoliosis Cobb angle was 140.3° ± 23.1°, which decreased to 94.8° ± 16.1° after treatment, resulting in an average scoliosis correction rate of 31.9%. The initial kyphosis Cobb angle was 109.2° ± 34.8°, which decreased to 82.6° ± 26.9° after treatment, resulting in an average kyphosis correction rate of 23.1%. During the second phase (from posterior release to spinal osteotomy), with a treatment duration of 130.3 ± 58.3 days, the Cobb angle for scoliosis further decreased to 71.1° ± 14.5°, with an average curve correction rate of 25.0%; the Cobb angle for kyphosis decreased to 66.2° ± 22.3°, with an average kyphosis correction rate of 19.5%. The average scoliosis correction rate during the overall treatment phase (from HPT to spinal osteotomy) was 48.9%, and the average kyphosis correction rate was 38.0%.

**Table 2:** Summary of baseline characteristics and postoperative follow-up results of patients in the two phases Data are presented as mean ± standard deviation.

Item	First phase	Second phase	Total phase
Treatment time	89.7±66.1	130.3±58.3	220.1±94.4
Initial Cobb angle (°)	140.3±23.1	94.8±16.1	140.3±23.1
Postoperative Cobb angle (°)	94.8±16.1	71.1±14.5	71.1±14.5
Average curve correction rate (%)	31.9	25.0	48.9
Initial kyphosis Cobb angle (°)	109.2±34.8	82.6±26.9	109.2±34.8
Postoperative kyphosis Cobb angle (°)	82.6±26.9	66.2±22.3	66.2±22.3
Average kyphosis correction rate (%)	23.1	19.5	38.0

Analysis of factors affecting correction outcomes

Fifty-four patients were divided into two groups based on the scoliosis correction rate (mean, 48.9%): a high-correction rate group (n=27, 50.0%) and a low-correction rate group (n=27, 50.0%). Chi-square tests and independent-samples t-tests were used to analyze differences between the two groups (Tables 3, 4). Significant differences were found in age (the high-correction rate group was younger, p=0.021). However, no significant differences were found between the two groups in sex (p=0.172), initial Cobb angle (p=0.175), treatment duration (p=0.091), scoliosis type (congenital or idiopathic) (p=0.761), comorbidities (rib fusion or spinal fusion) (p=0.074), or comorbidities (spinal cord malformation) (p=1.0). Further binary logistic regression analysis revealed that gender (p=0.032), age (p=0.011), and comorbidity (rib fusion or spinal fusion) (p=0.047) were risk factors affecting the scoliosis correction rate (Table 5).

**Table 3:** χ² test to assess differences between the two groups

	χ² value	degrees of freedom	p-value
Gender	1.86	1	0.172
Classification (congenital or idiopathic)	0.0923	1	0.761
Complications (rib fusion or spinal fusion)	3.20	1	0.074
Complications (spinal cord malformation)	0.00	1	1.000

**Table 4:** Independent sample t-test to assess differences between the two groups Hₐ μH ≠ μL

		Statistic	degrees of freedom	p-value
Age	Student's t value	-2.38	52.0	0.021
Initial Cobb angle	Student's t value	1.38	52.0	0.175
Treatment time	Student's t value	1.72ᵃ	52.0	0.091

**Table 5:** Binary logistic regression exploration of risk factors associated with scoliosis correction rate The coefficient represents the logarithmic ratio of "correction rate group = L" compared to "correction rate group = H"

Predictors	Coefficient	Standard Error	Z-value	p-value
Intercept	1.98940	3.12663	0.636	0.525
Gender				
Male-Female	1.78082	0.83061	2.144	0.032
Age	0.11691	0.04570	2.558	0.011
BMI	-0.03016	0.09717	-0.310	0.756
Initial Cobb angle	-0.03153	0.01893	-1.666	0.096
Treatment duration	-0.00983	0.00566	-1.737	0.082
Concomitant spinal cord deformity				
Yes-No	-0.59836	0.80084	-0.747	0.455
Concomitant rib fusion or spinal fusion				
Yes-No	1.95520	0.98510	1.985	0.047
Classification				
Congenital-Idiopathic	0.11654	0.98693	0.118	0.906

## Discussion

For patients with severe spinal deformity, direct osteotomy correction surgery carries the risk of serious complications, such as spinal cord injury and paralysis [18, 19]. Therefore, preoperative use of pelvic traction as a conservative and low-risk treatment method has important clinical value in improving the degree of scoliosis in patients [12, 20]. Studies have shown that HPT can significantly improve coronal and sagittal deformities before corrective surgery [21], and reduce postoperative complications, such as improving lung function in patients with scoliosis and reducing respiratory complications in patients after corrective surgery [22, 23], while also reducing the neurological complications of patients [24]. Our research shows that HPT can effectively reduce patients' Cobb angles for scoliosis and kyphosis. In the first phase of treatment, the average scoliosis Cobb angle decreased from 140.3° to 94.8°, with an average correction rate of 31.9%. At the same time, the kyphosis Cobb angle decreased from 109.2° to 82.6°, with an average improvement rate of 23.1%. This result is consistent with previous studies (e.g., Qi et al., who studied 30 patients with spinal deformity who underwent short-term preoperative cephalopelvic traction) showing a 30.24% correction rate for scoliosis and a 33.54% correction rate for kyphosis in the first two weeks [22]. In a study by Xu et al., the average scoliosis correction rate of 24 scoliosis patients who underwent primary HPT was 37.2% [25], The correction rate in this study was slightly lower than that reported in the literature. This discrepancy may be due to several factors: First, the patients included in this study had extremely severe scoliosis, with an initial Cobb angle of 140.3°, significantly higher than the patient population in other studies; second, some patients had complex medical conditions, such as spinal cord malformation or rib fusion, which may have had a certain impact on the surgical outcome.

**Figure 3:**
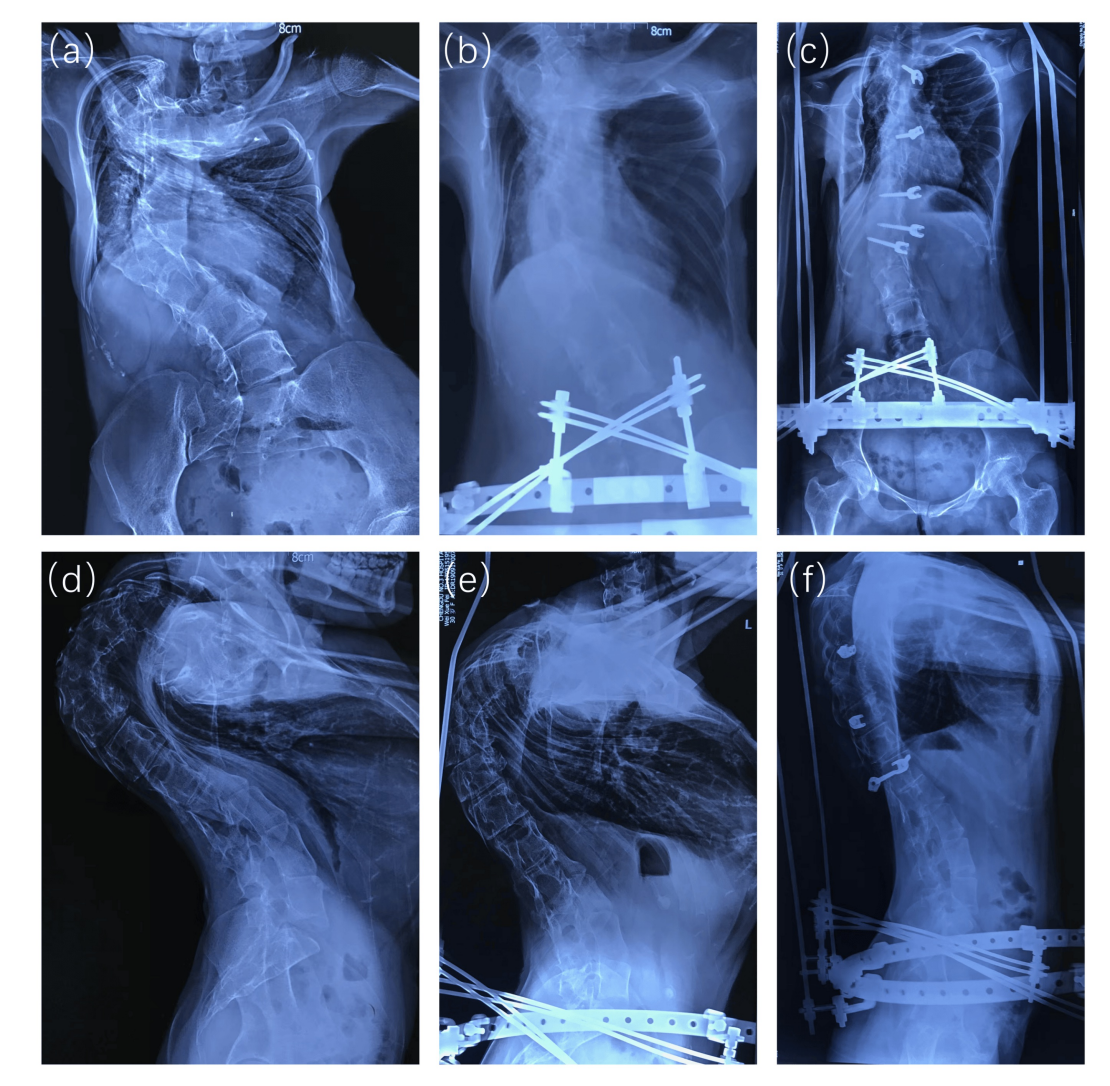
X-ray of a female patient undergoing HPT and posterior release surgery Figures 3a and 3d are X-rays taken upon admission, showing a scoliosis angle of 160° and a kyphosis angle of 142°. Figures 3b and 3e show that after 5 months of HPT, the scoliosis angle improved to 97° and the kyphosis angle improved to 118°. Figures 3c and 3f show that 3 weeks after HPT combined with posterior release surgery, the scoliosis angle and kyphosis angle further improved to 45° and 81°, respectively. Source: These radiographic images are from the patient enrolled in this study, who was admitted to and treated by the Department of Spinal Surgery, Chengdu Third People's Hospital.

Compared with HGT, HPT has shown greater applicability in the preoperative correction of severe scoliosis [12, 26]. However, there is still controversy about the optimal traction time for HPT. Excessive traction may lead to a series of complications, such as pelvic nail infection, cranial nerve and spinal cord injury, decreased spinal mobility and spontaneous cervical fusion [27-29]. To address the risk of these complications, we perform spinal release surgery on patients before corrective surgery. Compared with the anterior release surgery which may cause postoperative respiratory impairment and other complications [30-32], we prefer the posterior release surgery. Posterior release surgery further unlocks the spinal correction potential by relieving contractures and muscle spasms in the posterior spine. Our research has shown that combining HPT with posterior release surgery significantly improves the correction rate of scoliosis and kyphosis. Specifically, the average scoliosis Cobb angle decreased from 94.8° to 71.1°, the scoliosis correction rate in the second stage was 25.0%, and the total scoliosis correction rate reached 48.9%. The average kyphosis Cobb angle decreased from 82.6° to 66.2°, the kyphosis angle improvement rate in the second stage was 19.5%, and the total kyphosis angle improvement rate reached 38.0%. Compared with the study of 61 scoliosis patients by Zhou et al. (preoperative scoliosis Cobbangle of 114.2°±38°, kyphosis Cobb angle of 105.8°±34.7°), after a 19.2-week HPT traction, the scoliosis Cobb angle decreased to 55.3° and the kyphosis Cobb angle decreased to 52.6°. After the patients underwent posterior release surgery, the scoliosis angle was further reduced to 47.4° and the kyphosis angle was reduced to 38.1°, achieving an average correction rate of 58.3% and 49.5%, respectively [14]. Although our results differ from those in the literature, this is primarily due to the fact that the included patients had extremely severe spinal deformity (after one-stage HPT, the mean scoliosis and kyphosis Cobb angles were 94.8° and 82.6°, respectively), which are significantly higher than those reported in the literature. However, it is clear that the second-stage posterior release procedure further improved the patients' spinal deformity, confirming the effectiveness of a combined treatment strategy in patients with extremely severe deformity. Figure 3 shows a representative case of a female patient with severe spinal deformity who underwent HPT and posterior release.

In this study, we found that patient age was a significant risk factor for scoliosis correction (p = 0.011). As patient age increased, the scoliosis correction rate decreased significantly. This result is consistent with the general understanding in the field of scoliosis treatment: spinal flexibility is one of the key factors determining the effectiveness of scoliosis correction [33]. However, in this study, we are more concerned with the specific mechanism of age-related changes in this phenomenon. First, aging is often accompanied by the degeneration of the spine and its surrounding soft tissues. Many studies have shown that as patients age, spinal flexibility gradually decreases, leading to a decrease in surgical correction rates [34, 35]. This may be related to factors such as intervertebral disc degeneration, facet hyperplasia, and muscle and ligament stiffness. In this study, we observed that postoperative correction outcomes in elderly patients were significantly lower than those in adolescents. This trend suggests that early intervention in younger patients should be prioritized in clinical practice. Secondly, age has a significant impact on the sensitivity of scoliosis treatment. According to a study by Nascene et al. (2017), the optimal age range for surgery is generally 13 to 16 years old [35], as the bones at this age are not yet fully mature but still possess considerable plasticity and resilience. However, in this study, we found that even in patients outside this age range, age still significantly affected the rate of correction. This suggests that, in addition to considering spinal flexibility, the patient's age should be comprehensively assessed during preoperative evaluation.

Congenital scoliosis is usually caused by abnormal vertebral formation or segmental failure, which is often accompanied by rib fusion or spinal fusion [36]. For patients with congenital scoliosis, rib fusion or spinal fusion is often present [37]. Among the 54 patients with severe scoliosis in our study, 39 (72.2%）had congenital scoliosis, 32 of whom (82.1% of congenital cases）had concomitant rib fusion or spinal fusion. The postoperative correction rate in these patients was significantly lower than that in patients without such deformities. First, rib fusion and spinal fusion can lead to bone stiffness, which reduces the flexibility of the spine. This stiffness not only limits the effectiveness of corrective surgery but also increases the difficulty of surgery [38]. For example, the vertebrae in the fusion area usually lack a normal range of motion, making it difficult to achieve adequate correction during surgery. In addition, the scoliosis curves of these patients are often more complex and may include multiple curvatures or rotational deformities, further affecting the correction effect. Secondly, patients with combined rib fusion or spinal fusion may face more challenges during postoperative recovery. Due to bone stiffness, these patients may be more prone to postoperative complications such as failure of internal fixation or loss of correction. Therefore, for patients with combined rib fusion or spinal fusion, doctors need to formulate surgical plans more carefully and fully evaluate their potential risks before surgery.

In this study, we found that gender was a significant risk factor for postoperative correction rate in patients with severe scoliosis (p = 0.032). Male patients were significantly more likely to have a low correction rate than female patients. The impact of gender differences on spinal flexibility remains controversial. Studies have shown that during adolescence, girls typically have smaller vertebral cross-sectional areas than boys, which may contribute to greater spinal flexibility [39]. However, in adults, most studies have shown that women have a lower range of motion in spinal extension than men [40-42]. In this study, we observed a higher postoperative correction rate in female patients than in male patients. This may be related to several factors: First, the average age of the patients in this study was 26.1 years. Female patients in this age group may have relatively higher spinal flexibility. Gender differences during adolescence (such as a smaller vertebral cross-sectional area in girls) may be more significant in younger age groups. Second, gender differences are also related to the patient's growth and development pattern. Females generally enter puberty earlier, and their skeletal systems may reach maturity more quickly. This biological characteristic may enable female patients to demonstrate better stability and correction maintenance after surgery, thereby achieving better correction results.

Despite these findings, our study has several limitations. First, the lack of a control group is a constraint inherent to its single-arm design. Including a cohort of similar patients not undergoing preoperative Halo-pelvic Traction (HPT) was not feasible, both because HPT is the standard preparatory care for this specific severe patient population and due to ethical constraints, as withholding this treatment would be unjustified. Second, the sample size, while comprising 54 patients, remains relatively small for investigating extremely severe scoliosis, which may limit the generalizability and statistical power of our results. Third, the follow-up duration was insufficient to assess long-term outcomes due to the study design. Although short- to mid-term efficacy was demonstrated, the long-term maintenance of correction and potential complications requires evaluation through extended observation. Finally, the imaging measurements, though performed independently by two experienced physicians who then averaged the Cobb and kyphosis angles, are inherently subjective. The use of two-dimensional radiographs introduces potential measurement bias. Given these limitations, future research should focus on the following areas: Comparative Study Designs: Where ethically and clinically feasible, prospective (potentially multi-center) studies should compare the combined HPT and posterior release protocol with alternative preparatory strategies or different traction regimens in comparable patient populations. Long-term Follow-up: Studies with extended follow-up periods are needed to evaluate the durability of correction and the incidence of late complications. Individualized Treatment Plans: Developing personalized surgical and rehabilitation protocols based on patient-specific factors such as age, comorbidities (e.g., rib or spinal fusion), and gender could help improve correction rates and patient satisfaction. Advanced Imaging Assessment: Exploring and applying more objective three-dimensional spinal imaging techniques or artificial intelligence-assisted assessment systems could minimize measurement subjectivity and improve accuracy.

## Conclusions

This study demonstrates that HPT combined with posterior release can significantly improve spinal deformity in patients with severe scoliosis. Although HPT itself has limitations, such as its time-dependent nature, the combination with posterior release successfully overcomes this limitation and provides patients with more adequate preparation for subsequent spinal osteotomy. The results indicate that age, gender, and comorbidities (such as rib fusion or spinal fusion) are significant risk factors for scoliosis correction. These findings provide an important theoretical basis for further optimizing treatment plans and improving surgical outcomes.
